# The Relevance of a Physical Active Lifestyle and Physical Fitness on Immune Defense: Mitigating Disease Burden, With Focus on COVID-19 Consequences

**DOI:** 10.3389/fimmu.2021.587146

**Published:** 2021-02-05

**Authors:** Tayrine Ordonio Filgueira, Angela Castoldi, Lucas Eduardo R. Santos, Geraldo José de Amorim, Matheus Santos de Sousa Fernandes, Weydyson de Lima do Nascimento Anastácio, Eduardo Zapaterra Campos, Tony Meireles Santos, Fabrício Oliveira Souto

**Affiliations:** ^1^Keizo Asami Immunopathology Laboratory, Universidade Federal de Pernambuco, Recife, Brazil; ^2^Pós Graduação em Educação Física, Universidade Federal de Pernambuco, Recife, Brazil; ^3^Pós Graduação em Neuropsiquiatria e Ciências do Comportamento, Universidade Federal de Pernambuco, Recife, Brazil; ^4^Serviço de Nefrologia do Hospital das Clínicas, Universidade Federal de Pernambuco, Recife, Brazil; ^5^Núcleo de Ciências da Vida, Centro Acadêmico do Agreste, Universidade Federal de Pernambuco, Caruaru, Brazil

**Keywords:** COVID-19, social isolation, sedentarism, home-based exercise, immune system

## Abstract

The Severe Acute Respiratory Syndrome Coronavirus 2 (SARS-CoV-2) is a fast spreading virus leading to the development of Coronavirus Disease-2019 (COVID-19). Severe and critical cases are characterized by damage to the respiratory system, endothelial inflammation, and multiple organ failure triggered by an excessive production of proinflammatory cytokines, culminating in the high number of deaths all over the world. Sedentarism induces worse, continuous, and progressive consequences to health. On the other hand, physical activity provides benefits to health and improves low-grade systemic inflammation. The aim of this review is to elucidate the effects of physical activity in physical fitness, immune defense, and its contribution to mitigate the severe inflammatory response mediated by SARS-CoV-2. Physical exercise is an effective therapeutic strategy to mitigate the consequences of SARS-CoV-2 infection. In this sense, studies have shown that acute physical exercise induces the production of myokines that are secreted in tissues and into the bloodstream, supporting its systemic modulatory effect. Therefore, maintaining physical activity influence balance the immune system and increases immune vigilance, and also might promote potent effects against the consequences of infectious diseases and chronic diseases associated with the development of severe forms of COVID-19. Protocols to maintain exercise practice are suggested and have been strongly established, such as home-based exercise (HBE) and outdoor-based exercise (OBE). In this regard, HBE might help to reduce levels of physical inactivity, bed rest, and sitting time, impacting on adherence to physical activity, promoting all the benefits related to exercise, and attracting patients in different stages of treatment for COVID-19. In parallel, OBE must improve health, but also prevent and mitigate COVID-19 severe outcomes in all populations. In conclusion, HBE or OBE models can be a potent strategy to mitigate the progress of infection, and a coadjutant therapy for COVID-19 at all ages and different chronic conditions.

## Introduction

After decades of qualified research to understand the effects of physical inactivity and sedentarism on human health, this topic continues to be discussed and is still a challenge to achieve satisfactory levels of physical activity (1). The consequences of sedentary timeline evolution are continuous, progressive, unforgiving, and almost silent. A cyclic pattern is established, with no precise beginning, but includes: a reduction of corporeal capacity; an increase in physical and emotional discomfort when exposed to higher levels of physical demand; and a sedentarism behavioral pattern associated with any kind of exercise avoidance, which is often associated with others dangerous behaviors to health (2). The entrance to this vicious cycle might be promoted by social, economic, clinical, age, gender, schooling, race, civil status, and others factors (3). Despite these aspects, it is outside the scope of this review to explore these determinants.

The original silent aspect of sedentarism becomes abruptly relevant in suddenly adverse conditions such in war, public calamities, and pandemics, as that caused by the Severe Acute Respiratory Syndrome Coronavirus 2 (SARS-CoV-2) which resulted in Coronavirus Disease-2019 (COVID-19) (4, 5). Regardless of sedentarism, in calamity conditions, humans are exposed to mentally, physically, and nutritionally unusual situations, with a direct impact on health, mediated by, among other factors, the immune system (6).

SARS-CoV-2 infection compromises the immune system affecting individuals with underlying conditions or risk factors, such as cardiovascular and metabolic diseases on which a sedentary lifestyle can be a risk factor for morbidity and mortality (7, 8). Hypertension, type 2 diabetes, obesity, asthma, hematologic diseases, chronic obstructive pulmonary disease, chronic kidney disease, immunosuppression, and advanced age are associated with critical COVID-19 disease and higher hospitalization rates (9, 10). This raises interesting questions about the complex interaction between sedentarism and comorbidities, and how these impact COVID-19 outcomes, with special attention to the role of the immune system.

In order to discuss the close relationship between the patterns of physical activity and the consequent levels of physical fitness and the immune system, this review aims to outline objective considerations on the maintenance of physical activity that can balance the immune system and increase immune surveillance. In fact, many types of exercise, such as aerobic, strength, or combined training, and sports in indoor or outdoor environments can impact the immune response (11). Therefore, to promote potent effects against the consequences of chronic diseases associated with the development of severe forms of COVID-19. The present review discusses the benefits of physical activity, and special attention was given to home-based exercise (HBE) (exercise at home) and outdoor-based exercise (OBE) benefits as a powerful strategy to mitigate the progress of infection and adjunctive therapy for COVID-19 at all ages and different chronic conditions. HBE could be a potent strategy to maintain exercise levels regarding social distancing during quarantine periods, but also OBE can be a formidable strategy to reinforce the maintenance of physical activity for those who are comfortable to go out or who are transitioning to habitual exercise practice.

## Physical Health and the Impact of Associated Comorbidities on COVID-19 Severity

Clinical manifestations of COVID-19 arise after an incubation period of about 5.2 days, depending mainly on age (> 70 years), comorbidities, and on the efficiency of the immune response (12, 13). In summary, patient symptoms are reported as fever, cough, sore throat, respiratory dysfunction, SpO2 less than 95%, and fatigue, while other symptoms include sputum production, headache, hemoptysis, diarrhea, vomiting, dyspnea, acute myocardial injury, chronic cardiovascular damage, and lymphopenia (10, 14–16). These manifestations impact on physical health, which can provide a rapid evolution of the clinical picture. This dramatically increases the risk of mortality from associated comorbidities (17).

The clinical spectrum of SARS-CoV-2 infection ranges from asymptomatic to critical illness. From the documented cases of COVID-19, 80% of individuals are asymptomatic or experience mild symptoms to moderate disease (18). Approximately 10-15% of the cases progress to severe disease and about 5% become critically ill (18, 19). The estimation of susceptibility to infection in individuals under 20 years of age is approximately half that of adults over 20 years old, and clinical symptoms manifest in 21% of infections in 10 to 19-year-olds and rise to 69% of infections in people over 70 years old (20). In fact, the mortality rate is also higher in elderly individuals. A systematic review, which included 113 studies, observed an exponential relationship between age and the infection fatality rate for COVID-19. The estimated age-specific fatality for this rate is very low for children and younger adults (0.002% at age 10 and 0.01% at age 25), but increases progressively to 0.4% at age 55, 1.4% at age 65, 4.6% at age 75, and 15% at age 85 (21). This reinforces the need for public health measures to mitigate severe infection in older adults that could substantially decrease the hospitalization and mortality rates.

Confinement reduces the level of physical activity, muscle mass, and function, increasing the risk of metabolic and health complications, especially in the elderly (22–24). This scenario can anticipate a clinical picture of sarcopenia, the condition already demonstrated to be related to increased fragility and risk of falls, which provide a higher incidence of negative health outcomes, including cardiovascular and respiratory disorders, chronic obstructive pulmonary disease, and a high mortality rate (24–26). Pre-sarcopenia, sarcopenia, and reduced muscle strength are associated with acute respiratory distress syndrome (ARDS) and death (27), which can be related to the impaired immune response in COVID-19 patients (28). However, more studies are necessary to understand the mechanisms behind the relationship between sarcopenia, fragility, social isolation, and hospitalization.

Increased COVID-19 severity in hypertensive individuals is possibly associated with the involvement of the renin-angiotensin-aldosterone system, which has a role in the pathology of COVID-19 infection (29). In diabetic patients, uncontrolled glycemia is a major risk factor for the severity and mortality of COVID-19 (30). Hyperglycemia directly induces SARS-CoV-2 replication and proinflammatory cytokine production (31). SARS-CoV-2-infected monocytes present higher glycolytic activity with increased mitochondrial ROS production, which induces stabilization of hypoxia-inducible factor-1α (HIF-1α) and consequently promotes glycolysis. This was shown to be necessary for viral replication. These changes induced by HIF-1α in monocytes upon SARS-CoV-2 infection directly inhibit T cell response and reduce epithelial cell survival (31). Increased blood glucose is a risk factor for COVID-19 severity, however, a better comprehension of the impact of diabetes and insulin treatment will provide more information about the mechanisms behind the severity of COVID-19 patients, and in diabetic nephropathy where angiotensin-converting enzyme 2 (ACE2) circulating activity seems to be higher in both the early and late stages of the disease (32). In addition, most of these patients are under treatment for their previous comorbidity and it might account for the COVID-19 outcomes. Many of these drugs are immunomodulatory compounds and it impacts on the immune response.

There is a dose-response relationship between physical activity and health outcomes that provides a reduction in the risk of mortality related to the aforementioned aggravating factors (33, 34), raising the hypothesis of better outcomes and decreasing hospitalization rates related to COVID-19 disease in physically active patients. Below, we discuss in more detail the effects of physical activity on immune response and chronic diseases.

## COVID-19: Immune Response

COVID-19 disease was highlighted as a new beta-coronavirus pandemic, with a high risk of mortality and contagion presented in its mild, severe, or critical form. Phylogenetic analysis shows that SARS-CoV-2 is part of the Coronaviridae family, such as SARS-CoV and MERS-CoV, similar pathogens previously reported to cause severe respiratory disease in humans (35). The genomic correlation between SARS-CoV-2 to SARS-CoV and to MERS-CoV is 82% and 50% of genetic similarity, respectively (36). The morphology of SARS-CoV-2 consists of single-stranded positive RNA and the transmembrane spike glycoprotein (S protein).

The S protein has been shown to promote the virus connection with the ACE2 of the host with ten to twentyfold greater affinity than SARS-CoV or MERS-CoV (37). This enzyme is found mainly in the epithelial cells of the respiratory tract, such as alveolar type 2 (AT2)-epithelial cells. The infection is first established in pneumocytes and enterocytes of the small intestine and also infects the airway club and ciliated cells of the upper respiratory tract (38). Although, there is evidence of SARS-CoV-2 infection in tongue keratinocytes (39). Moreover, ACE2 is observed in the kidney and heart tissue, gastrointestinal tract, and blood vessels, which can induce critical complications as well (40, 41).

**Figure 1:**
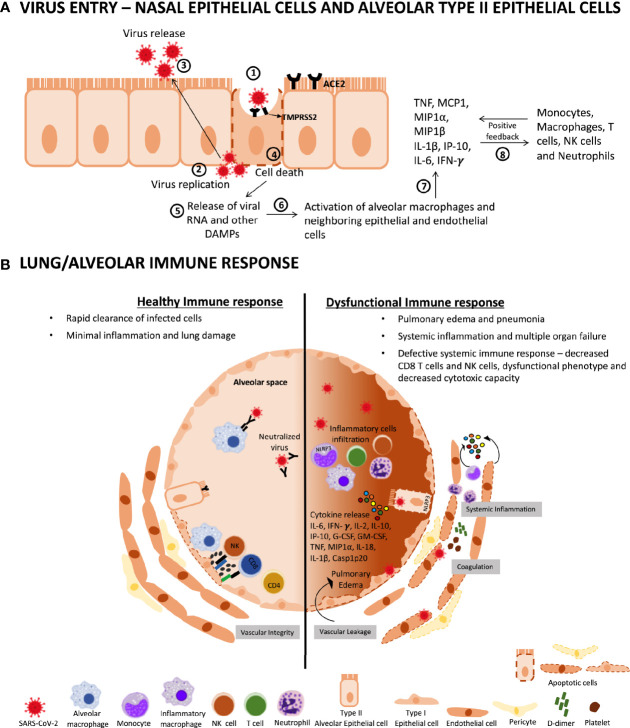
Inflammatory responses during SARS-CoV-2 infection. **(A)** Virus entry – nasal epithelial cells and alveolar type II epithelial cells: (1) when SARS-CoV-2 infects cells expressing the surface receptors ACE2 and TMPRSS2, (2) the active replication and (3) release of the virus induces (4) the death of host cells. (5) These cells will then release DAMPs, including ATP and viral RNA which will be (6) recognized by neighboring epithelial cells, endothelial cells, and alveolar macrophages. (7) Proinflammatory cytokines and chemokines (including IL-6, TNF, IL-1β, IP-10, MIP1α, MIP1β, and MCP1), will be generated by those cells. (8) These proteins attract monocytes, macrophages, T cells, and NK cells (which will secrete IFNγ) to the site of infection, promoting further inflammation. **(B)** Lung/alveolar immune response: in a healthy immune response (left side), alveolar macrophages recognize neutralized viruses and apoptotic cells and clear them by phagocytosis. The initial inflammation attracts virus-specific T and NK cells to the site of infection, where they can eliminate the infected cells before the virus spreads. In addition, neutralizing antibodies can block viral infection and CD4 T cells mediate efficient immune response. Altogether, these processes lead to clearance of the virus and minimal lung damage, resulting in recovery and maintaining vascular integrity. In a dysfunctional immune response (right side), this may lead to further accumulation of immune cells (massive infiltration of monocytes, T cells, NK cells macrophages, and neutrophils in the lungs) causing overproduction of proinflammatory cytokines, which eventually damages the lung structure leading to pulmonary edema and pneumonia. Moreover, the NLRP3 inflammasome is induced by SARS-CoV-2 in monocytes and epithelial cells, which will result in cell death and the release of IL-18, IL-1β, and Casp1p20. The resulting cytokine storm circulates to other organs, leading to multi-organ damage and defective systemic immune response, with decreases CD8 T cells and NK cells as well as decreases cytotoxic capacity. Moreover, SARS-CoV-2 affects vascular epithelial cells leading to endotheliitis development, and consequent dysfunction and death of endothelial cells. In addition, accumulation of immune cells and inflammatory cytokines might lead to loss of inter-endothelial junctions which contribute to increased vascular permeability and vascular leakage. The increased vascular leakage leads to pulmonary edema. Moreover, activation of the coagulation pathway with the accumulation of D-dimers and secretion of inflammatory cytokines by both epithelial cells and immune cells contribute to the systemic inflammatory response observed in severe/critical COVID-19 patients. ACE2, angiotensin-converting enzyme 2; DAMPs, damage-associated molecular patterns; IL, interleukin; G-CSF, granulocyte colony-stimulating factor; GM-CSF, granulocyte-macrophage colony stimulating factor; IP-10, interferon gamma-induced protein-10; MIP1, macrophage inflammatory protein 1; TNF, tumor necrosis factor; NLRP3, NLR family pyrin domain containing 3; NK, natural killer cell; Casp1p20, Caspase-1 subunit p20.

SARS-CoV-2 infection of AT2 epithelial cells also depends on type II transmembrane serine protease (TMPRSS2) activity (42). The virus attaches to the ACE2 receptor, and the spike protein is cleaved by the TMPRSS2 resulting in viral replication (42). SARS-CoV-2 then infects AT2 epithelial cells leading to cell death (43–46). The death of infected cells releases viral RNA (45) and damage-associated molecular patterns (DAMPs) such as ATP and DNA (47, 48) which will result in the activation of alveolar macrophages (49) and neighboring epithelial cells, culminating in the secretion of the proinflammatory cytokines interleukin (IL)-1β, tumor necrosis factor (TNF), IL-6 (46, 50), and the chemokines monocyte chemoattractant protein (MCP)-1, macrophage inflammatory protein (MIP)1-α, MIP1-β, and human interferon-inducible protein 10 (IP-10) (19) (**Figure 1**).

This initial inflammation attracts monocytes, macrophages, and virus-specific T cells and natural killer (NK) cells to the site of infection, where they eliminate the infected cells before the virus spreads (49, 51). Neutralizing antibodies can block viral infection (52), and alveolar macrophages recognize neutralized viruses in dying cells and clear them by phagocytosis (53). Altogether, these lead to the clearance of the virus and minimal lung damage, resulting in recovery (**Figure 1**). However, in cases of an impaired immune response, the infection spreads preferentially to the lower respiratory tract (54, 55), leading to the severe outcomes of the COVID-19 disease (**Figure 1**).

In parallel, monocytes, macrophages, and adaptive immune cells such as CD8 T cells and CD4 T cells, are mobilized to the respiratory tract which contributes to IFN-γ secretion, promoting further inflammation and establishing a proinflammatory response (54, 56). Upon reaching the bloodstream, it takes seven days for the viral load to increase due to the reduction of systemic immune cells, mainly NK and CD8 T cells (51). The decreased number of NK cells and CD8 T cells in the peripheral blood in addition to marked cell exhaustion was associated with increased expression of CD94/NK group 2 member A (NKG2A). NKG2A levels have been shown to induce NK and CD8 T cell exhaustion in chronic viral infections. The increased expression of NKG2A in COVID-19 patients was associated with a decreased secretion of IFN-γ, IL-2, and granzyme B from both NK and CD8 T cells (51), suggesting that the impaired immune response during SARS-CoV-2 is due at least in part to exhausted NK and CD8 T cells. Moreover, the amount of key antiviral mediators, type I and III IFN, is diminished during SARS-CoV-2 infection, which is in contrast with what is seen in other viral infections (50, 57). This suggests an immunomodulatory effect to SARS-CoV-2 infection, which leads to increased systemic viral load.

The factors that trigger severe illness in individuals infected with SARS-CoV-2 are not completely understood. However, the development of severe disease does not seem to be only related to viral load and can be associated with the defect in the type I IFN response by T cells (19). Furthermore, during the course of COVID-19, severe inflammation is established through the aggressive production of proinflammatory cytokines, known as cytokine release syndrome (58). Due to the cytokine storm, high levels of proinflammatory cytokines circulate to other tissues, establishing multi-organ damage, with severe and critical COVID-19 exhibiting considerably increased serum levels of proinflammatory cytokines including IL-6, IL-1β, IL-10, IL-2, IL-8, IL-17, granulocyte colony-stimulating factor (G-CSF), granulocyte-macrophage colony-stimulating factor (GM-CSF), IP10, MCP-1, MIP1-α, IFN-γ, and TNF, which is thought to be the major cause of disease severity and death in COVID-19 patients (56) (**Figure 1**).

NLR family pyrin domain containing 3 (NLRP3) inflammasome activation was also shown to contribute to the exacerbated inflammatory response in COVID-19 patients. Active NLRP3 was found in PBMCs and postmortem tissues from moderate and severe COVID-19 patients. Inflammasome-derived products such as Casp1p20 and IL-18 in the sera were correlated with the markers of COVID-19 severity, including IL-6 and LDH. Higher levels of IL-18 and Casp1p20 are associated with disease severity and poor clinical outcome (59). These, associated with increased levels of other proinflammatory proteins lead to shock and tissue damage in several organs such as the heart, liver, and kidney, and the development of respiratory or multiple organ failures (19, 60, 61) (**Figure 1**).

The inflammatory events observed during COVID-19 disease reinforces the crucial role of an effective immune response during SARS-CoV-2 infection that depends on the release of inflammatory molecules during the disease and shows the importance of immune cells, especially T cell response to control SARS-CoV-2 infection and the consequent development of severe COVID-19.

Given that the morbidity and mortality seen in COVID-19 are mainly associated with excessive inflammation, failure in the adaptive immune response leading to an increased viral load, and organ failure, a better comprehension of what drives these events upon SARS-CoV-2 infection is necessary to better identify therapeutic targets and the best time to start the treatment. Moreover, associated comorbidities are often related to severe COVID-19, and it is not clear whether the impaired immune response is a result of associated diseases or if it is a characteristic of the SARS-CoV-2 infection that might evade the immune system.

## COVID-19: Physiopathology

Due mainly to an impaired immune response, approximately 15% of COVID-19 cases become severe/critical and progress to severe pneumonia and about 5% develop ARDS, septic shock, and/or multiple organ failure (19) which are often associated with the presence of comorbidities/risk factors described before. The disease progression associated with an excessive and dysregulated inflammatory response may cause harmful tissue damage at the site of virus entry and at the systemic level. This excessive proinflammatory response has been described to induce an immune pathology presented as complications in bilateral pulmonary parenchyma and pulmonary opacities (62), severe pneumonia resulting in acute lung injury, and ARDS in severe COVID-19 patients (60) and requires artificial ventilation (19). Clinical and laboratory findings in patients with COVID-19 pneumonia include micro-hematuria, proteinuria, and acute kidney injury (AKI). The presence of AKI has been shown as an important systemic complication in severe cases of COVID-19 worldwide and was associated with higher mortality (63). Mechanisms in which SARS-CoV-2 affects the kidneys are multifactorial, and case series and retrospective studies reported mechanical ventilation and hypotension requiring vasopressors as risk factors for AKI (63). Cheng and Luo (64) have reported an increase in serum creatinine and blood urea nitrogen, and a decrease of estimated glomerular filtration. Hence, these complications were associated with the hospital-death of COVID-19 patients (64).

Besides that, SARS-CoV-2 has a high tropism for the kidney where it has been shown to replicate in almost 30% of COVID-19 patients (65) once renal parenchyma is rich in ACE2 receptors expression (66). Postmortem series cases were described as direct proximal tubular cells and podocytes infection (65, 67). Severe acute tubular necrosis with lymphocyte and macrophage infiltration, prominent acute proximal tubular injury, and peritubular erythrocyte aggregation and glomerular fibrin thrombi with ischemic collapse were also observed in these patients (65, 68). Thrombophilia might be associated with the evolution of acute tubular necrosis to cortical necrosis, and hence, nonreversible kidney injury (69). However, some studies have reported that most hemodialysis patients might be likely to experience mild disease which does not develop into full-blown pneumonia, probably due to the reduced function of the immune system and decreased cytokine storms (70).

Immune hyper-response and hyperinflammation, besides that previously mentioned, induce generalized endothelial damage, contributing to increased coagulation, endotheliitis, and systemic microangiopathy (71). In fact, coagulation abnormalities, lower platelet counts, and increased levels of fibrin degradation products such as D-dimers have been shown to be associated with poor prognosis and could represent the main cause of organ failure and death in patients with severe COVID-19 (72, 73). As coagulation imbalance is a characteristic of sepsis, mediated by proinflammatory cytokines (74), it is possibly the trigger for the same events in COVID-19 patients. Besides the effects on organ failure, deregulated blood coagulation functionality is associated with the cardiovascular and nervous systems (75, 76). In this sense, microvascular thrombosis was found in patients with COVID-19 (77). This condition is often associated with heart dysfunction, including tachycardia, bradyarrhythmia, and acute myocardial infarction (77, 78). Although the neurological impacts have not been properly studied, patients with COVID-19 may present symptoms at the neurological level, whether nonspecific, such as headaches and confusion, or specific, such as seizure or cerebrovascular problems (79). They have been justified because SARS-CoV-2 is not only restricted to the respiratory tract but also invades the central nervous system (80). In the circulatory structure of the nervous system, COVID-19-induced inflammatory response must provide devastating effects through hypercoagulability, induced by sepsis, which can cause imbalances in proinflammatory and vasoconstricting effects in a large proportion, resulting in irreversible brain damage, including stroke (76, 81). However, anticoagulant therapy appears to be associated with a better prognosis in severe COVID‐19 patients (82).

In fact, COVID-19 disturbs several systems leading to severe outcomes in a considerable portion of the affected population. However, the growing incidence of long-term effects of COVID-19 opens a discussion about the consequences of this disease and suggests that longer-ranging longitudinal observational studies will be critical to elucidate the durability and complexity of the health consequences of COVID-19 (83).

## COVID-19: Skeletal Muscle Manifestations

Clinical manifestations of skeletal muscle involvement have also been described during SARS-CoV-2 infection, not only in currently infected or recovering patients but also in uninfected individuals during quarantine and lockdown recommendations (84, 85). Mild muscle symptoms like myalgia and muscle weakness were reported in one-quarter to one-half of COVID-19 patients (19, 86), and more severe symptoms, such as myocardial injury, with an elevation in cardiac damage biomarkers (troponin I and creatine kinase-MB (CK-MB) (87) and rhabdomyolysis have also been described (88). Moreover, it is known that the deleterious effect of hypomobility caused by isolation with restriction to daily activities that require commuting and rest is associated with COVID-19 symptoms. Evidence has shown a significant decline in cardiorespiratory fitness, induction of insulin resistance, vein thrombosis, and muscle atrophy due to prolonged bed rest (89, 90). Therefore, this scenario, the reduction of physical stimuli due to hospitalization or lockdown, might boost the side effects of COVID-19.

The skeletal muscle system is a major organ in human body and represents almost 40% of the corporal mass in adults. It is responsible for the force generation that allows for corporal movement execution and daily activities (91). Skeletal muscle is a highly organized tissue formed by bundles of myofibers (muscle fiber) that contains several myofibrils. Each muscle fiber has a functional unit called sarcomere which is responsible for muscular contraction (92).

The maintenance of skeletal muscle mass depends on the balance between the mechanisms that promote increased synthesis and/or the degradation of proteins in the myocyte (93). Factors capable of altering this equilibrium can cause muscle mass loss and sarcopenia (94). The regulation of protein synthesis in muscle is attributed mainly to the insulin-like growth factor 1-Akt/mammalian target of the rapamycin (IGF1-Akt/mTOR) pathway and to satellite cells. First, when stimulated by factors such as diet, exercise, and anabolic hormones such as IGF-1 and testosterone, is able to promote increased synthesis and the inhibition of muscle protein catabolism pathways (95). Second, because there are stem cells present in the sarcolemma of the muscle fiber, they are activated by the injury of the cell, caused by both physical exercise and trauma, and participate in the repair processes of muscle tissue (96).

The activation of intracellular pathways of ubiquitin-proteasome and myostatin (92, 97), by factors such as inflammation, angiotensin 2, and glucocorticoids disuse, induces the regulation of atrogen expression, muscle ring finger 1 (MuRF-1) and atrogin-1, responsible for the breakdown of muscle proteins (98).

**Figure 2:**
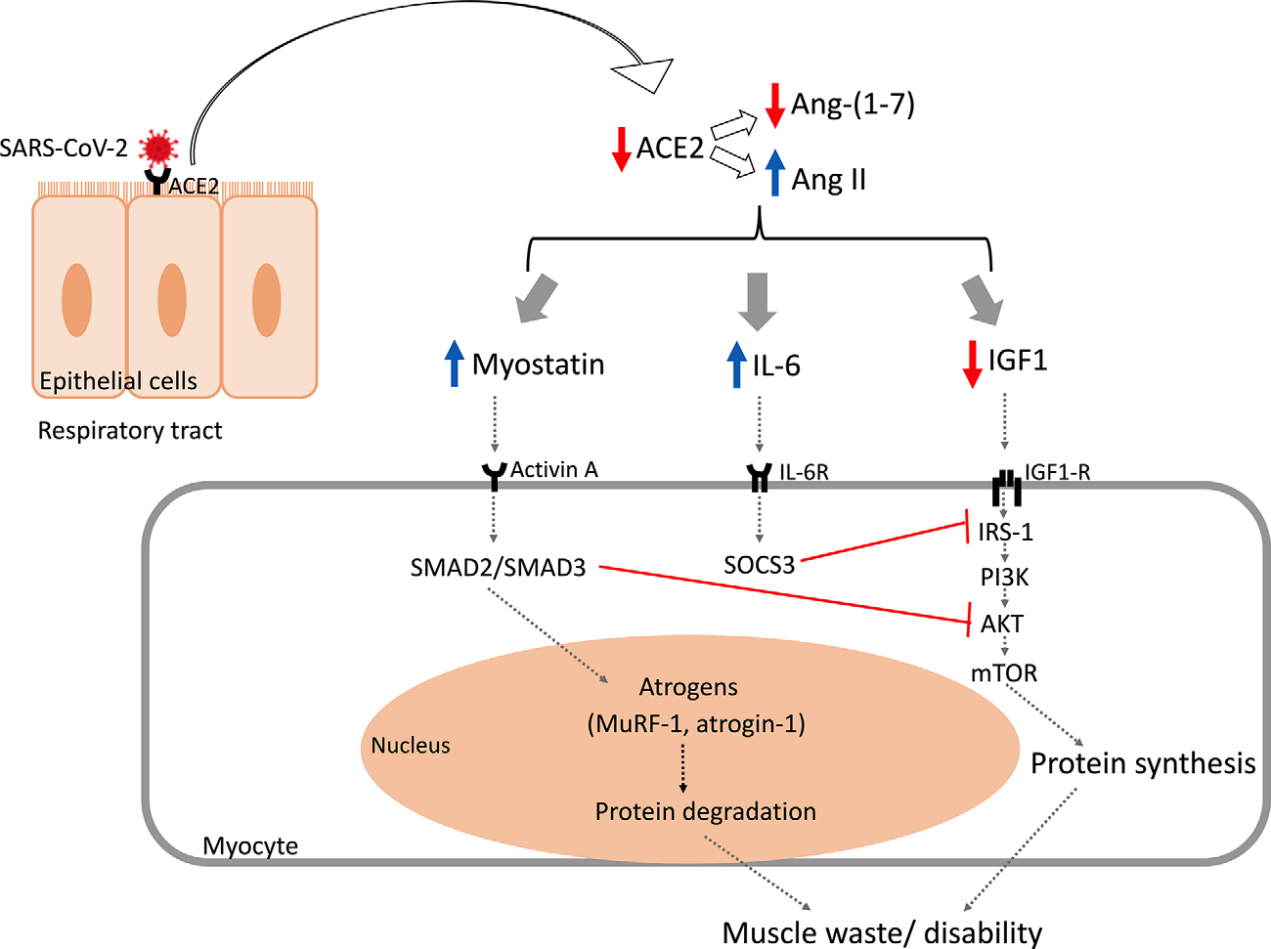
Mechanisms by which SARS-CoV-2 promotes muscle wasting and sarcopenia. A proposed mechanism is attributed to the direct viral infection, given that ACE receptors are widely expressed in skeletal muscle, especially in satellite cells. Another mechanism is attributed to the interaction between SARS-CoV-2 and the ACE2 receptor in respiratory epithelial cells that diminishes the biodisponibility of the ACE2 receptor. This promotes a deregulation in the renin-angiotensin-system with elevation in Ang II and decreasing in Ang 1-7. Ang II leads to increased levels of myostatin and IL-6 and decreased IGF-1 expression. Myostatin modulates the intracellular signaling molecules SMAD2/SMAD3, leading to increased expression of atrogens, such as MuRF-1 and atrogin-1, and inhibits PI3K/Akt/mTOR signaling. Moreover, increased IL-6 expression plays a role through SOCS3 which also inhibits the IGF1-R signaling pathway. Together, these activities promote increased protein breakdown and decrease protein synthesis in muscle, which leads to muscle wasting and disability. Ang, angiotensin; MuRF-1, muscle ring finger 1; IGF1, insulin-like growth factor 1; IGF1-R, insulin-like growth factor 1 receptor; PI3K, phosphoinositide 3-kinase; Akt, protein kinase B; mTOR, mammalian target of rapamycin; SOCS, suppressor of cytokine signaling.

Several mechanisms have been proposed to explain the occurrence of wasting and muscular dysfunction in infected SARS-CoV-2 patients. A proposed mechanism is attributed to the direct viral infection, given that ACE receptors are widely expressed in skeletal muscle, especially in satellite cells, mesenchymal stem cells, endothelial cells, and lymphocytes (99). Besides that, it was proposed that SARS-CoV-2 could promote dysregulation in the renin-angiotensin-system with elevation in angiotensin II (Ag-II) and a decrease in angiotensin 1-7. Elevation in Ag-II and its interaction with the angiotensin-1 receptor leads to augmented inflammation, and pro-fibrotic and pro-apoptotic events in skeletal muscle (85). The mechanisms by which SARS-CoV-2 promotes muscle wasting and sarcopenia are described in **Figure 2**.

The hyper inflammation and cytokine storm, common in COVID-19 patients, with an elevation in IFN-γ, IL-1β, IL-6, IL-17, and TNF, promotes muscular proteolysis, decreased protein synthesis, and satellite cells dysfunction, possibly another mechanism of muscle wasting in these individuals (100, 101). Likewise, corticosteroids therapy (102) and mechanical ventilation, commonly used in intensive care unit (ICU) patients (85), and physical inactivity, due to public health recommendations for quarantine (5, 103), are other factors associated with the occurrence of skeletal muscle wasting and sarcopenia during the COVID-19 pandemic.

Special regard is given to populations with a high prevalence of myopathies like the elderly and dystrophic muscle carriers, like Duchenne dystrophy, where SARS-CoV-2 infection could promote worsening of muscle wasting, sarcopenia, and frailty (100, 104, 105). After SARS infection, patients could present with altered muscle tests and disability for a long period, and low strength, muscle mass loss, and worsening in physical performance have been described as long as three months after hospital discharge (106). These findings suggest that physical and nutritional support are fundamental for COVID-19 patient rehabilitation, especially for the elderly.

COVID-19 has been established as a systemic disease with mild, severe, or critical respiratory illness associated with an important increase in inflammatory cytokines serum levels and commitment of the immune system that plays an essential role in SARS-CoV-2 infection. In this context, a traditional active lifestyle might promote a complex set of benefits to mitigate the deleterious effects of SARS-CoV-2 infection on the immune system and, therefore, it must improve the outcome of COVID-19 in individuals affected by comorbidities.

## Acute and Chronic Effects of Exercise on the Immune Defense

A sedentary lifestyle and a low level of physical fitness are associated with increased plasma levels of proinflammatory cytokines, such as IL-1β, IL-6, IL-7, TNF, and C-reactive protein (CRP) (107–109). Concomitantly, high chronic levels of systemic proinflammatory cytokines have been associated with a higher incidence of metabolic, cardiovascular, and rheumatic diseases, in addition to type 2 diabetes (110–112).

In contrast, exercise is an effective therapeutic strategy to mitigate a series of metabolic disorders (113). Besides the effect on metabolic changes, exercise must take out potent effects against the “cytokine storm syndrome” experienced in COVID-19 patients. Indeed, studies have shown that an acute stimulus of physical exercise induces myokines production, such as myostatin, IL-6, IL-7, IL-8, IL-10, IL-15, and leukemia inhibitory factor, which are secreted in the tissues and in the plasma, supporting the systemic modulatory effect of physical exercise (11). In this context, physical exercise was found to improve cell-mediated and humoral immunity, promoting enhanced immunosurveillance with latent therapeutic value to the consequences of infections and chronic diseases (114). Other studies have suggested that exercise can significantly increase antibody responses to vaccination, mainly in patients with a low immune defense (115, 116). Besides, exercise can prevent the incidence of viral infections, the time of the infection, and mortality by viruses, such as influenza, rhinovirus, and herpesviruses (117, 118).

The acute exercise-induced immune response depends on exercise characteristics, such as type, intensity, duration (119), and its interaction with the subject’s fitness level. Acute exercise is viewed as an efficient stimulus to improve CD34^+^ hematopoietic stem cells and mobilize cell-mediated immunity, such as a two- to fivefold increase in the blood circulating leukocytes (120). Exercise bouts have been reported to raise the mobilization of NK cells, enhance neutrophil chemotaxis and phagocytosis, and the modulation of inflammatory/alternative activated macrophages in adipose tissue (121, 122). Further, a proportion of broad proliferation and migration of adaptive immunity cells was found due to acute exercises, such as viral-specific CD4 and CD8 T cells, and discrete responses in memory T cells. High levels of effector cells reach the bloodstream, lung, intestine, and lymphoid tissues, which in this context are able to defend the organism against external infectious agents, such as SARS-CoV-2 (122). In addition, acute exercise may be related to the improvement of humoral immunity due to the production of myokine by muscle contraction due to muscle regeneration (11). These findings suggest the importance of exercise since these immune cell subtypes are closely linked to viral response, such as the response to SARS-CoV-2.

Chronically, the effects of sessions of exercise will modulate immune system plasticity and might result in decreased chronic inflammation and inducing resistance against infections and chronic diseases (123). In the infection scenario, the benefits of chronic exercise on immune defense have been shown. In this context, the myokines, IL-6, and IL-8 promote immune effector cell traffic, such as leukocytes, monocytes, neutrophils, and T cells by adhesion molecules and chemokine receptors expression (124, 125). Moreover, the myokines IL-7 and IL-15, involved in T cell homeostasis, promote the maintenance of immune defense in tissues, such as the lungs, against infectious agents (11). In addition, physical exercise has been associated with the improvement of immune defense by decreasing fat tissue that promotes the enhancement of low-grade chronic inflammation (126).

One of the proposed mechanisms is through the secretion of myokines that improve glycolytic and lipolytic metabolism (127). These metabolic effects were demonstrated by better glycemic control and weight loss in diabetic and nondiabetic patients (128, 129). Besides that, weight loss which is potentialized by chronic physical exercise decreases chronic low-grade inflammation observed in obese individuals (130, 131). Furthermore, the practice of physical activity has been associated with promoting immunity maintenance, potent systemic anti-inflammatory action, contributing to prevent cardiometabolic diseases (132).

Together, these studies have shown the beneficial effects of exercise on several aspects of the immune response and suggest that exercise training, which is associated with an efficient immune defense and better glycemic control which is shown to play a role in immune cell activation by SARS-CoV-2 (31) would be an effective strategy against hospitalization rates induced by viral respiratory diseases, such as COVID-19.

## Effects of Exercise-Mediated Immune Response on Respiratory Diseases

Respiratory infections have been associated with acute and chronic changes in cell-mediated immunity, promoting increased systemic inflammation. In this sense, an impaired immune system can be considered as a mechanism for the development of the severe disease, and adjunctive therapeutic strategies, such as physical exercise, would prevent inflammation, balance the immune system, and increase immune vigilance. In this context, studies have looked at the effects of exercise on the modulation of the immune response and decreasing rates of infections, such as in viral diseases (133). Whereas few studies have shown that high-intense and prolonged (strenuous) exercise causes the suppression of immune defense and progressively increases the risk of infections [“J-curve”, first reported by Nieman (134)], to demonstrate a relationship between physical fitness and immune function (135). Additionally, acute febrile infections have been associated with low performance during exercise due to muscular atrophy, circulatory disturbance, and impaired motor coordination. Acute symptoms of infection, such as COVID-19, warrant caution until the nature and severity of the disease is known (136). Therefore, strenuous exercise during viral infections and fever may be risky, but also should be avoided until, at least, the symptoms have normalized and the infection has ended. However, other factors were implicated in the impairment of immune response by physical exercises, such as age, infection history, mental stress, sleep disruption, and nutrition (137). Together these findings reinforce the side effects of extreme exercise before, during, or after viral infection associated with immunosuppression and substantial morbidity and mortality (138, 139).

Generally, randomized controlled studies have reported the impact of moderate regular exercise to decrease the incidence of upper respiratory tract infection (URTI) (140). Other studies have demonstrated a reduction of around 50% of the symptoms of URTI as a result of chronic exercise. Besides the characteristics of exercise, the incidence of URTI might be impacted by physical fitness. In this regard, studies have related a decrease of around 28% in the incidence of URTI rates in people with high fitness and physical activity levels. Moreover, these studies have reported a decrease of symptoms and, mainly, a reduction in the severity of URTI, decreasing the number of days of URTI in 43% and 46% in people who practice moderate and high fitness capacity, respectively (135). Experimental research has shown that higher cardiorespiratory fitness may improve lung function and have anti-inflammatory properties (141). These effects may be in part explained by the upregulation of ACE receptors in the lungs, with augmented angiotensin 1-7 production. The vasodilator, antiproliferative, and antifibrotic effects of angiotensin 1-7 production could mitigate the deleterious effects of COVID-19 in the lungs (141).

In parallel, the impact of exercise on influenza has already been investigated. An experimental study reported regular beneficial effects of moderate exercise on the inflammatory response and reduced viral load (142). Investigational studies have shown that physical exercise can inhibit lung inflammation and pneumonia by bacterial colonization (143, 144). Furthermore, a clinical study with the elderly has shown that moderate or intense training can be a modifying factor in stronger and longer-lasting antibody responses to the influenza vaccine (145). Moreover, the positive effects of both chronic and acute exercise on vaccine-induced immunogenicity were observed, and seroprotection against influenza A/H1N1 and A/H3N2 (116, 146) was significantly associated with chronic exercise. Evidence has also been reported that people who practice exercise can have decreased respiratory tract infection incidence, but also could reduce the breathlessness in the postoperative patient (147).

During the COVID-19 course, an increasing number of studies have shown mild or severe cytokine release syndrome, characterized as a cytokine storm, in patients who develop a severe disease (148). IL-6 is an important member of the cytokine network and plays a central role in acute inflammation, and it has been shown to be one target for treatment. However, it has been known for a while that IL-6 is a pleiotropic molecule (149). IL-6 is released into the blood during exercise and it exerts anti-inflammatory effects in several disease models (150). However, adaptation to exercise training must reduce IL-6 production and counterbalance potential stimulation for IL-6 secretion (151). It has been shown that voluntary running protects aged mice against exacerbated sepsis-induced inflammatory response in the lung. This effect was associated with decreased IL-6 and neutrophil infiltration and increased endothelial nitric oxide synthase protein in the lung after sepsis (152). The opposite was observed during influenza infection, IL-6 knockout mice had increased mortality associated with reduced macrophage infiltration in the lung, increased fibroblast proliferation, decreased epithelial cell survival, and increased collagen deposition, suggesting a role for IL-6 in regulating fibrosis development (153, 154). These findings suggest that IL-6 is required for protection against fibrosis development secondary to influenza infection, however, physically active patients, adapted to regular exercise could be protected from the severe outcome of COVID-19 by anti-inflammatory effects of exercise adaptation, which is also associated with decreased IL-6.

## Effects of Exercise-Mediated Immune Response to Chronic Diseases

Several studies have established the effect of physical exercise to prevent chronic diseases, but mainly, as an efficient therapy to treat chronic diseases (155). The immune system has been highly recognized as the link between these areas. Chronic diseases such as obesity, type 2 diabetes, cardiorespiratory diseases, many cancer types, psychological disorders [e.g., depression (156), anxiety (157), bipolar disorder (158), and post-traumatic stress (159)], and neurodegenerative diseases [e.g., Alzheimer’s (160) and Parkinson’s (161)] are characterized by high levels of oxidative stress, serum systemic proinflammatory cytokines, and immune dysfunction. The positive impact of regular exercise in chronic degenerative diseases is summarized by its ability to increase cell-mediated humoral immunity, systemic anti-inflammatory myokine production, regulation of transcription factors expression, and molecular changes in weight loss (162). Moreover, exercise is associated with increased systemic levels of antioxidant enzymes, including catalase (around 28%), superoxide dismutase (74.5%), and glutathione peroxidase (41%) (163). Along with that, low levels of these enzymes are linked to several diseases, such as atherosclerosis, Parkinson’s disease, and Alzheimer’s disease (164, 165), suggesting that regular physical exercise could be beneficial and improve disease outcomes. Therefore, intensity-moderated exercise appears to have a complete health-promoting effect involving the regulation of redox and inflammatory homeostasis when compared to physical inactivity groups (166).

The role of physical exercise was also shown to improve T cell responses, increasing both NK and CD8+ T cell mobilization into the blood, migration into the tissues, and TNF activation of these cells (122, 167, 168), and this is known to be the mechanism by which physical exercise confers protection against cancer (169). In this regard, we would speculate that regular physical activity might mitigate the development of severe COVID-19 disease since increasing the activation of NK and CD8 T cells would improve the immune response against SARS-CoV-2.

The impact of physical exercise has also been strongly reported as a regulatory mechanism for the immune system in obesity (170). One way is through the reduction of adipose tissue (171). It is well known that hypertrophy and hyperplasia of the adipocytes generate a hypoxic environment which is the trigger for the increased inflammation observed in the adipose tissue during obesity. It begins with apoptosis of adipocytes and secretion of inflammatory factors that will then recruit monocytes that further differentiate into classical inflammatory macrophages, increased secretion of proinflammatory cytokines (such as TNF, IL-6, and IL-1β), and infiltration of other immune cells, including the adaptive immune cells CD4 and CD8 T cells (172). In this context, evidence has been reported that moderate exercise decreases classical inflammatory markers in blood monocytes and reduces proinflammatory chemokines (such as C-C chemokine receptor (CCR)-5, Rantes, C-C motif chemokine ligand (CCL)-2, and intracellular adhesion molecule (ICAM)-1) (173). In addition, low-intensity exercise is associated with an anti-inflammatory systemic profile characterized by increased expression of genes related to alternative activation of macrophages (174, 175).

Moreover, physical exercise is shown to enhance insulin sensitivity (127) and this effect is linked to IL-6 (176), which together with IL-10 and IL-1Ra are able to reduce IL-1β signaling (177), and consequently decrease insulin resistance. Further, high levels of muscle-derived IL-6 have been reported to improve glucose uptake by AMP-activated protein kinase-dependent pathways (127).

On the other hand, dysregulated continual production of IL-6 leads to the onset or development of various diseases. It induces the synthesis of acute-phase proteins such as CRP, serum amyloid A, fibrinogen, and hepcidin in hepatocytes, whereas it inhibits the production of albumin (178). IL-6 also plays an important role in the acquired immune response by stimulation of antibody production and of effector CD4 T cell development and inhibits TGF-β-induced regulatory T cell (Treg) differentiation (179, 180). During the severe stage of COVID-19, the inhibition of the IL-6 receptor (IL-6R) is being tested in clinical trials, and it has shown promising results in decreasing the cytokine release syndrome in COVID-19 patients (148). It was shown recently (181) in a randomized controlled trial with abdominally obese individuals who received an IL-6R blocking antibody (tocilizumab) or placebo during a 12-week intervention with either bicycle exercise or no exercise that the effect of exercise in reducing visceral adipose tissue was abolished in the presence of IL-6 signaling blockade. This suggests that IL-6 signaling is required for the beneficial effects of exercise. Although the effects of exercise training-secreted IL-6 on immune response modulation are well reported (148), more data are needed in humans for a set of severe inflammatory diseases in order to ensure the safe practice of exercise to mitigate or to treat patients at risk of developing severe COVID-19.

A meta-analysis of randomized controlled trials established the effectiveness of exercise in reducing the main clinic conditions of type 2 diabetes, such as hemoglobin A1C and insulin resistance (182, 183). The beneficial effects of exercise to treat obesity-related conditions were also associated with decreased IL-6 and TNF observed in trained obese individuals.

Moreover, several studies have established the effect of regular physical activity to prevent and contribute to the treatment of cardiovascular diseases. Immunoregulation of chronic inflammation is also one pathway by which exercise helps the cardiovascular system, vessel tissues, and intima-media thickness of arteries, such as the carotid (184). Another important benefit of regular exercise is to improve the serum levels of antioxidant molecules, promoting fibrinolytic activity, and preventing thrombogenesis (185). Besides this evidence, future studies are needed to confirm the effects of exercise in immunosurveillance to viral infections in people with chronic diseases.

It is not known clearly yet if there is further chronic disease development after COVID-19 infection. However, physical exercise could be offered to patients recovering from COVID-19 in order to prevent lung fibrosis complications. Studies performed in SARS survivors showed that these patients had mild impairment of their lung function three months to two years after hospital discharge and most of the patients had decreased physical exercise capacity (186, 187). Another study showed that a 6-week supervised exercise training program in patients recovering from SARS was effective in improving both cardiorespiratory (VO_2Max_ (3.6 mL/kg/min vs 1 mL/kg/min, *p* = 0.04) and musculoskeletal fitness (188). This study suggests that supervised exercise training could improve lung function in patients recovering from SARS-CoV-2.

The studies mentioned here and in the previous sections are detailed in **Table 1**.

**Table 1:** Summary findings of the effects of acute and chronic exercise protocols in immune response.

	Type of study	Methods					Findings
		Sample	Exercise protocol				
			Type	Frequency	Intensity	Duration	
Philips et al. (113)	Cross-sectional study	396 adults (ages 50–69 years)	General physical activities	7 consecutive days	Moderate to vigorous physical activity	–	30 min of the physical activity was associated with higher adiponectin and lower complement component C3, leptin, IL-6 and white blood cells concentrations. In obese subjects, moderate to vigorous physical activity was associated with lower white blood cells concentrations.
Edwards et al. (115)	Randomized controlled trial	133 young healthy adults	Resistance training	1 session	Sets of 30 seconds of exercise and 30 seconds of rest	15 min per session	Exercise increased antibody levels. Therefore, these results indicate the effectiveness of exercise as a vaccine adjuvant.
Williams (117)	Cohort study	109,352 runners and 40,798 walkers	Aerobic training	–	–	–	Higher doses of running and walking have decreased the respiratory disease mortality in 7.9% per MET-hour per day and 7.3% for all respiratory disease-related deaths. Pneumonia mortality decreased 13.1% per MET-hour per day, but also 10.5% per MET-hour per day for all pneumonia-related deaths.
Diment et al. (119)	Experimental	64 healthy and recreationally active males	Aerobic training	1 session	60% V˙ O2peak (30MI); 80% V˙ O2peak (30HI); 60% V˙ O2peak (30MI)	30 min per session; 30 min per session; 120 min per session	Immune induction by DPCP was impaired just by 120 min per session group.
Mobius-Winkler (120)	Experimental	18 healthy young men	Aerobic training	1 session	70% of their individual anaerobic threshold.	240 min	A significant increase in leukocytes, as a very early rise in vascular endothelial growth factor and later increase in IL-6. All observed changes were normalized 24 hours after finishing the test.
Campbell et al. (122)	Experimental	13 healthy and physically active males with age 20.9 ± 1.5 years old	Aerobic training	–	35% Wattmax (low intensity exercise); and 85% Wattmax (high intensity exercise)	20 min	High intensity exercise induced strong differential mobilization of CD8TL subsets that exhibit a high effector and tissue-migrating potential (RAEM > EM > CM > naïve). Increased NK cells mobilization attributed to increased CD56dim NK cells.
Nieman et al. (135)	Observational study	1002 adults (ages 18–85 years, 60% female, 40% male)	Aerobic training	–	–	12 weeks	The number of days with URTI was significantly reduced, 43% in subjects reporting daily aerobic exercise compared to those who were largely sedentary and 46% when comparing subjects with low fitness routine. The URTI severity and symptomatology were also reduced 32% to 41% between high and low aerobic activity routine.
Matthews et al. (140)	Observational study	547 healthy adults (49% women) aged 20–70 years old	Moderate-vigorous activity	–	–	–	The risk of URTI event was reduced by about 20% in men and women.
de Araujo et al. (145)	Cross-sectional	61 healthy elderly men with 65-85 years	Sports, and Aerobic running (Volleyball, Basketball or Running)	≥ 5 days/Week	Moderate, and Intense	Maintenance of active lifestyle for 5 years	Subjects who practiced moderate or intense physical training had long-standing antibody responses to the influenza vaccine components, resulting in higher percentages of seroprotection.
Bhatt et al. (147)	Prospective case control study	30 cases and 30 case matched controls aged 18 years or more	Aerobic training	2 days (Acute exercise)	Moderate (≥ 70% of max heart rate)	10 minutes per session	The practice of early aerobic activity with a pedaler halves the rate of respiratory tract infection and postoperative hospitalization after complex abdominal surgery. In addition, in the subjective shortness of breath, there was also a reduction with the use of a pedal exerciser, meaning the potential to improve resistance to exercise in the postoperative patient.
Tyml et al. (152)	Animal study	Male C57BL/6 mice	Voluntary running wheel	1-3 days per Week	High levels of voluntary physical activity	8 weeks	Voluntary running has been able to protect against exacerbated sepsis induced by inflammatory and pro coagulant responses in aged mice. These were due to increased eNOS protein after running exercise.
Bigley et al. (168)	Randomized Trial	Twelve male and four female trained, and non-smoking cyclists	Aerobic training	1-3 Weeks	Incremental protocol with power variation 5% to 15%	Continuous cycling for 30 minutes	Physical exercise was able to promote a preferential redistribution of subsets of NK cells with a highly differentiating phenotype and increases cytotoxicity against HLA expression target cells.
Baturcam et al. (173)	Experimental study	Adult male and female nondiabetic subjects	Aerobic and resistance training	3 to 5 times per Week	Moderate (50 - 60% of max heart rate)/Vigorous (65 - 80% of max heart rate)	3 months	Physical exercise significantly decreased expression of RANTES and CCR5 in adipose tissue of obese patients with concomitant reduction in TNF, IL-6, and p-JNK.
Yakeu et al. (174)	Experimental study	17 healthy adults	Aerobic training	3 times per Week	Low intensity (10.000 steps/week)	8 weeks	Physical exercise was associated with the positive regulation of markers linked to the function of M2 macrophages, PGC-1α and PGC-1β. However, it negatively regulated the functionality of the M1 macrophage markers. In addition, plasma levels of Th2 cytokines increased after exercise, while those of Th1 cytokines decreased.
Barry et al. (175)	Randomized controlled trial	37 inactive obese 30–65 years old	High Intense Interval Training (HIIT) and Moderate Intensity Continuous Training (MICT)	5 times per week	–	2 weeks (equivalent to 10 sessions)	Moderate-intensity continuous training decrease percentage of monocytes positives for receptor C-C motif chemokine receptor, reduced surfaced protein expression of C-X-C chemokine receptor on monocyte, in addition high intensity interval training increased protein expression and percentage CCR5 positive monocytes, T cells and neutrophils.
Wedell-Neergaard et al. (181)	Randomized controlled trial	53 patient samples (27 exercise and 26 non-exercise)	Aerobic training	3 times per Week	High intensity interval training (50 - 85% of VO_2max_)	12 Weeks	Physical exercise reduced visceral fat. The effect of exercise was abolished in the presence of IL-6 blockade. Blocking IL-6 increased cholesterol levels, an effect not reversed by physical exercise. Therefore, IL-6 is necessary for exercise to reduce visceral fat tissue mass and emphasizes a potentially important metabolic consequence of IL-6 blockade

IL-6, Interleukin 6; MET, Metabolic equivalent of task; DPCP, Diphenylcyclopropenone; RAEM, CD45RA+ effector-memory; EM, Effector memory; CM, Central memory; NK, Natural Killer; URTI, Upper respiratory tract infection; eNOS, Nitric oxide synthase 3; HLA, Human leukocyte antigen; CCR5, C-C chemokine receptor type 5; TNF, Tumor Necrosis Factor; p-JNK, c-Jun N-terminal kinase; PGC-1α, Peroxisome proliferator-activated receptor-gamma coactivator 1 alpha; PGC-1β, Peroxisome proliferator-activated receptor-gamma coactivator 1 beta; CCR5, C-C chemokine receptor type 5.

## Effects of Exercise on Immunosenescence and Potential Impact on COVID-19 Severity

Immunosenescence contributes substantially to decreased health in the elderly as well as to increased systemic inflammation during aging, termed inflammaging. Inflammaging has been related to an increased risk of most chronic diseases in the elderly (189). Indeed, the influence of an active lifestyle on health in old age may lie in its impact on inflammaging, as regular physical activity has been associated with reduced systemic inflammation in older adults (190, 191).

Advanced age is associated with remodeling of both the innate and the adaptive immune response, which can lead to compromised immunity and disease. The key changes include decreased migration and antimicrobial function in neutrophils (192) and monocytes (193), reduced quality and quantity of antibody production by B cells (194), reduced NK cell cytotoxicity (195), thymic atrophy (196), increased frequency of T cells with senescent/exhausted phenotype (197), and increased secretion of proinflammatory cytokines and chemokines (198) by senescent cells. Moreover, increased coagulation and fibrinolysis activity in the elderly (199) are associated with a higher risk of age-related diseases, such as cardiovascular disease (200).

The effects of physical activity on immunosenescence remain unexplored. However, few studies approach this topic by assessing immune cell phenotypes in physically active individuals who have maintained physical activity during adulthood. A study performing analysis on healthy males (18-61 years old; *n* = 102), observed a positive correlation between aerobic fitness (VO_2Max_) and the frequency of naïve T cells and reduced levels of senescent/exhausted CD4 and CD8 T cells (201). Moreover, increased frequency of naïve T cells and recent thymic emigrants in cyclists compared with inactive older adults was also observed by another study comparing immune profiles in 125 adults (55-79 years) who had maintained a high level of physical activity (cycling) during their adult lives; 75 age-matched older adults and 55 young adults not involved in regular exercise (202). The active individuals also had significantly higher serum levels of IL-7, known to be thyme protection, and lower IL-6, which promotes thymic atrophy, compared to age-matched inactive older adults. Besides, cyclists also showed additional evidence of reduced immunosenescence, such as Th17 polarization, and higher B regulatory cell frequency than inactive controls. However, this study did not observe changes in the frequency of senescent/exhausted CD8 T cells in physically active compared to inactive elderly people (202).

Taken together, these studies suggest that physical activity could mitigate some features of immunosenescence, and consequently improve the immune response. However, the effects of immunosenescence on immune response against SARS-CoV-2 are not well explored. Lymphopenia occurs in over 80% of COVID-19 patients with marked reductions in circulating levels of CD4, CD8 T cells, and NK cells (51, 203), which suggests that immunosenescence would worsen this scenario. Associated with that, decreased phagocytic capacity by senescent resident macrophages may promote a proinflammatory state and impair phagocytosis of infected epithelial cells, where an imbalanced aged immune response is then exacerbated by COVID-19 (43–46). Moreover, the effects of immunosenescence on the quality and quantity of antibodies directly affect the immune response against SARS-CoV-2, eventually leading to harmed virus neutralization, increased viral load, and severe COVID-19 (52). In addition to that, the systemic low-grade inflammation in elderly individuals might play a role in the cytokine storm observed in severe COVID-19 patients (55). Besides, coagulation defects observed in immunosenescence might also increase COVID-19 severity, since coagulation imbalance is a feature of severe COVID-19 (72), impacting the mortality rate. In this scenario, the need for physical activity becomes necessary in order to prevent the effects of immunosenescence and to mitigate the evolution of COVID-19 disease as well as hospitalization rates.

Data constantly updated from Our World in Data (204) show that the numbers of COVID-19 confirmed cases per one million inhabitants in countries in which the percentage of older adults is higher than 25% of the total population, such as in Japan (1,339.61) and the European countries: Italy (29,277.13), Greece (11,486.07), and Germany (14,971.34) are not as high as in countries such as United States (46,484.71) and Brazil (31,654.45). It is therefore suggested that the high rates of confirmed cases do not appear to be associated with the high prevalence of elderly people in these countries, however, the incidence of death and hospitalization rate in the elderly is higher compared to younger adults and a more active lifestyle by this population might mitigate those consequences (21). It is possible that other aspects influence the incidence of cases in the elderly population, such as lifestyle, socioeconomic status, and public policies to mitigate contamination (205, 206).

In fact, physical inactivity is recognized as a major cause of the development of chronic diseases (207), in which high levels of inactivity can lead to greater damage to the immune system, especially in the elderly. These statements seem to be in agreement with the prevalence of physically inactive individuals in the countries with the highest incidence of cases. High-income Western, Asian Pacific, and Latin American countries are among the most physically inactive worldwide (208), according to Guthold and Stevens (208) which analyzed 1.9 million individuals from 168 countries and showed that globally, more than a quarter of adults (27.5%, 95% UI 25.0–32.2) were insufficiently physically active in 2016 (208). The prevalence of insufficient physical activity ranged from 36.8% (95% UI 34.6-38.4) in high-income Western countries, 35.7% (95% UI 34.4-37.0) in high-income Asian Pacific, and 39.1% (37.8–40.6) in Latin America and the Caribbean. Brazil was found to be one of the countries with the highest prevalence of inactivity at 47.0% (38.9–55.3) (208).

Together these data suggest that the prevalence of the elderly population is not directly associated with the high prevalence of COVID-19 positive cases, however, inactivity levels seem to play a role.

Corroborating, it was shown recently by an observational study that the performance of at least 150 min per week of moderate physical activity and/or 75 min per week of vigorous physical activity reduces the prevalence of hospitalizations by 34.3% in a group of 938 Brazilian survivors and those completely recovered from COVID-19 from all ages after adjustment for age, sex, BMI, and preexisting diseases (209). This study shows the importance of physical activity to mitigate the consequences of COVID-19, however, the mechanisms behind this associative effect still have to be clarified, although the beneficial effects of physical activity on the immune system have a potential role in the observed outcome.

## Physical Activity Recommendations to Improve the Immune System

In order to maintain physical fitness levels during social distancing periods, protocols to maintain exercise practice are suggested. The American College of Sports Medicine (ACSM) has been consolidating the ideal recommendations, as type, frequency, duration, and intensity to promote health benefits by physical exercise to all age groups (210, 211). The recommendation based on exercise characteristics is known to be associated with different immune responses and immune surveillance, especially the intensity (212). Since exercise became an important way to maintain a healthy immune system and its benefits embrace individuals of all ages, it is important to adapt the practice to each age group and existing chronic conditions in order to avoid injuries and side effects.

**Table 1** summarizes the effects of physical activity protocols on the immune response. It is important to note that moderate exercise improves several aspects of the immune system in both young and elderly individuals (from 18 to 85 years old). As described in previous sections, the beneficial effects of physical activity are broad and range from a decreased inflammatory profile, modulation of immune cells mobilization to decreased URTI infections, and increased antibody levels. Both short and longer physical exercise practice induced benefit effects. However, there is evidence that long periods of high-intensity exercise for long periods seem to induce immunosuppression and may be associated with later increased proinflammatory cytokines. Furthermore, a period of at least two weeks (Short-term Chronic Exercise) of physical activity is sufficient to positively stimulate the immune system, modulating cell migration and cytokine secretion, and inducing long-term effects. On the other hand, acute exercise sessions induce acute effects on the immune system, which return to basal levels shortly afterward. The observed long-term effects of physical activity on the immune system are linked to improved quality of life and decreased severity of several disease states, including COVID-19. In general, the studies described in **Table 1** reinforce the potential benefits of physical activity in promoting immune defense.

Accordingly, to the ACSM, a good way to promote health in different clinical conditions is to adapt the exercises following moderate intensity (40%-59% of reserve heart rate or oxygen uptake, 12-13 of Ratings of Perceived Exertion, and 50%-69% of one maximum repetition), no less than 30 min of duration and at least five days a week with professional support (213). In addition, 20 min of vigorous physical activity (60%-89% of reserve heart rate or oxygen uptake, 14-17 of Ratings of Perceived Exertion, and 70%-84% of one maximum repetition) at least three days a week, besides staying active throughout the day with daily activities was recommended (213).

To attain the recommended target, an option is to combine moderate and vigorous physical exercise corresponding to a demand of ≥ 500 to 1000 metabolic equivalent of task (MET)-minutes per week (considering that 1 MET corresponds to the consumption of 3.5 mL of oxygen for each kilogram of body mass per minute) which correspond to ≥ 5400–7900 steps/day or approximately 4-6 km (213). The previous recommendations characterized the individuals as ‘active’ if they reached the minimum of 10,000 steps/day, which corresponds to approximately 7.5 km (214). However, more recently, it was established that a number > 7500 steps/day (5.7 km) seems to be enough for individuals to achieve the recommended daily energy expenditure (215). As shown by Tudor-Locke and Craig (215), it seems prudent to use the number of steps to identify the level of sedentary physical activity and behavior. However, some limitations need to be considered regarding the characteristics of the investigated populations, the instruments used to quantify the steps, and the variety of body movements that are not characterized as steps, but have a fundamental role in the daily energy expenditure (215).

The recommendations mentioned above are based on the performance of aerobic activities, however, the ACSM also suggests the implementation of routines to maintain or increase muscular strength and endurance for at least two days a week (213). Ideally, individuals should train each major muscle group for a total of 2-4 sets with 8-12 repetitions and rest intervals between sets of 2-3 min and between sessions of 48 h (213). The recommended intensity to improve muscular strength ranges from 60% - 70% of one maximum repetition (1-RM) in moderate intensities and ≥ 80% of 1-RM in vigorous intensities (213). In addition, the ACSM also recommended at least two days a week for a series of flexibility exercises for each major muscle-tendon group (holding static for 10-30 s) and neuromotor activities to improve balance, agility, coordination, and gait for 20-30 min per day (213).

Also, the following recommendations for preventing undesirable effects of exercise in people with pulmonary and respiratory diseases suggest that exercise should be a mandatory component of pulmonary rehabilitation (213). Regarding the individuals with asthma, volume and exercise type are similar to the usual recommendations, but it is suggested that they make progressive and slight increases in intensity, beginning with moderate intensities and if well-tolerated progress to 60%-70% of reserve heart rate (213). These recommendations could be achieved in different facility contexts, including HBE with appropriate adequacy and creativeness to build a viable exercise program.

On the other hand, an excessive increase over a long period of training load has been described as a variable that negatively impacts immune cells. As previously discussed, the practice of prolonged exhaustive exercise was associated with an increase in the risk of common infectious diseases. Additionally, few studies have related the practice of prolonged intensive exercise as the cause of acute immunosuppression (201), like the immunodeficiency “window”, increasing the odds of illness, such as COVID-19 (132). Evidence has suggested that increased levels of anti-inflammatory cytokines during intense and prolonged exhaustive exercise, might lead to an increased risk of the development of URTI (216). Therefore, practicing exercise in closed environments and crowding might favor the transmission of viruses and should be avoided (217). Future studies and guidelines should explore this subject.

In parallel, a point to be considered is the season, especially winter, which reverberates in the immune system, increasing the risk of infectious diseases (135, 216). As well as the temperature change which affects energy supply to the immune system, decreasing the immune surveillance, and consequently, greater exposure to upper respiratory tract-related diseases (218, 219).

## Home-Based Exercise and Outdoor-Based Exercise

The World Health Organization (WHO) guideline recommends at least 150 to 300 min per week of moderate or 75 to 150 min of vigorous-intensity exercise per week, besides a combination of both, including aerobic resistance, and flexibility exercise protocols to all adults, including the elderly and patients with chronic health conditions or disability, but also recommends an average of 60 min per day for children and youth to reduce levels of physical inactivity and improve global health (220). During the COVID-19 pandemic and the wide recommendation for the temporary interruption of different service providers, including health and fitness facilities, HBE has been shown as a possible strategy for people to stay active and end sedentary behavior in social isolation scenarios or with limited options. The need to maintain the exercise routine can be supplied by HBE which might help to reduce levels of physical inactivity and sitting time, impacting on adherence to exercise and attracting patients in different stages of treatment for chronic diseases (221–223) due to its convenience. In addition, WHO highlights that HBE must overcome environment limitations during all the stages of the COVID-19 lockdown, providing a cheap, safe, and controllable exercise protocol to the general population with all ages whether they are affected by COVID-19 or not (224).

Moreover, few studies have shown improvements in cardiorespiratory capacity, blood pressure control, and improvements in the quality of life in practitioners of HBE (225, 226). A meta-analysis of randomized controlled trials also showed improved cardiorespiratory capacity, decreased fatigue, and reduced symptoms of adjuvant treatment in breast cancer survivors (227). In a sedentary group, another study has shown that after 24 weeks of HBE, subjects had better levels of metabolic assets, such as reducing fasting blood glucose, glycated hemoglobin, total cholesterol, and triglycerides, decreased fat tissue, blood pressure, and proinflammatory hs-CRP serum levels. In addition, it was observed that high levels of high-density lipoprotein (HDL) cholesterol (228). In the elderly population, a systematic review and meta-analysis reported the effect of HBE to reduce falls, sarcopenia, delaying cognitive health, and dementia, and also improve balance, mobility, and muscle strength (229, 230).

In addition to the benefits described, through the application of HBE, studies have shown that this activity can significantly improve the immune system. In the pneumonia scenario, HBE in frail elderly people was reported to enhance peak cough flow (p<0.01) and physical function (p<0.05), preventing aspiration pneumonia (231). Moreover, in thyroid cancer patients, 12 weeks of HBE promoted a significant enhancement in the levels of NK cells (232). Other cancer randomized controlled trials have observed a significant reduction of TNF serum levels after 12 weeks of HBE (233, 234). In stage II-III colorectal cancer survivors, HBE increased levels of insulin-like growth factor-1, IGF binding protein (IGFBP)-3, and adiponectin (233). Lately, some evidence has noted an enhancement of HDL levels after 12 weeks of HBE, but also these results were associated with decreasing IFNγ serum levels in postmenopausal women (235).

In this context, it is known that HBE with a self-selected prescription template must be efficient to improve cardiorespiratory capacity and peak oxygen consumption (236). The characteristics of the self-selected model present evidence that points to a direction for participants to experience moderate exercise intensities (237). Thus, it is possible that, if properly prescribed, this strategy would become efficient in promoting the maintenance and/or development of physical fitness in recovered COVID-19 patients and individuals in quarantine, to the detriment of the coronavirus. The self-selected activities model is also characterized by prioritizing improvements in affective aspects related to the participants’ pleasure (237) and the importance of affective responses in adhering to physical exercise has already been demonstrated (238). Instructions could be obtained by mobile applications, specific hornbooks, online videos, or preferably, by presential or remote supervision by a certified professional (235).

Although highly recommended, especially in the context of social isolation, the practice of HBE is not characterized as the only way to avoid physical inactivity. Another option is the practice of physical activity in places where it is possible to maintain a safe distance from others, so called outdoor based exercises (OBE). OBE is characterized by the number of physical activities done in outdoor spaces, for example in green places, green gyms, parks, squares, beaches, or neighborhood outdoor spaces. The findings by Lahart and Darcy (239) do not suggest better physical or wellbeing benefits in indoor vs. outdoor activities. However, it is possible that the practice of activities in open environments, with immersion in nature, such as parks, fields, and streets can have a positive impact on the maintenance and development of physical fitness and mental health (239). Kim and Lee (240) have observed that six weeks of combined exercise using outdoor exercise equipment was effective in enhancing fitness capacities, such as reduction of retinoic acid receptor responder protein 2 levels and insulin resistance in the elderly. Moreover, a randomized controlled trial has remarked a decrease of physiological stress by cortisol levels due to walking in nature (241). In another study, it was found that in this moment of isolation, individuals are more likely to travel long distances to be in urban green spaces, where exercising and relaxing seems to be the most desired activities (242). Therefore, it is suggested that practicing exercise in compliance with hygiene and safety standards is fundamental regardless of the environment.

It is important to highlight that the social isolation caused by the pandemic implies a series of negative consequences for mental health. A recent systematic review by Vindegaard and Benros (243) showed increased levels of post-traumatic stress and depression after infection. In addition, indirect negative effects were observed, such as increased symptoms of anxiety, depression, and insomnia, both among patients and health care workers (243). Another concern that must be considered by public authorities is in vulnerable families who may be victims of domestic violence and multiple types of aggressive and depressive behavior (244), which in this moment of social isolation can become more present, especially in groups more vulnerable to psychosocial stressors (245).

In the context of depression, the symptoms seem to be associated with lower amounts of muscle mass among adults and the elderly, and this may be a consequence of levels of physical activity, an association between muscle fitness, functional limitations and disabilities, frailty, and health-related quality of life (246). In fact, it is known that exercise is crucial to reducing the deleterious effects related to muscle mass loss caused by aging or a sedentary lifestyle (247). Muscle mass loss naturally causes decreases in muscle strength, which in turn can be associated with mental health, especially in people with depressive symptoms. In a review by Marques and Gomez-Baya (246), it was suggested that muscle fitness is inversely related to depressive symptoms among adults and older adults. It is possible that individuals with reduced muscle strength may feel less comfortable and motivated to exercise, such as HBE or OBE. Future studies should try to confirm the possible influences that muscle mass loss and strength can exert in adherence to physical exercise programs, and in future behavior.

Interventions focused on short term exercises with potential for physical and psychological improvements should be used as strategies to mitigate the effects of reduced muscle strength and demotivation to exercise. One of the possibilities, with low cost and few limitations, may be in the handgrip exercise. In a review by Rijk and Roos (248), associations were described between handgrip strength and cognitive, functional, and mobility status, suggesting that low levels of handgrip strength can predict the decline of these conditions. The effectiveness of handgrip strength exercise as a predictor of mood responses, depression, vigor, and sleep quality in elderly women has also been demonstrated (249). Therefore, it is suggested that this type of exercise may have viable potential to promote adequate physical and psychological responses, especially in populations limited in terms of possibilities to perform physical exercise and in terms of health and physical fitness.

Together, all the consequences described need to be taken into account and discussed, leading to a better prescription of physical exercises. Each physical exercise proposal, whether HBE or OBE, must be directed to mitigate the symptom presented by the individual, whether due to muscle mass loss, strength reduction, or depressive state. For example, being outdoors can be a relief in an emotional state negatively impacted by confinement measures. In fact, loneliness has already been shown to have significant effects on moderate levels of depression (250). And according to Torales and O’Higgins (251), global health measures must be taken to meet the demands that impact the psychosocial aspects related to isolation. In summary, HBE and OBE can have important differences that must be considered before practicing, since the external environment can generate positive impacts on mental health, in addition to improving the immune defense. It is therefore suggested that physical exercise could be seen as a way to promote physical and mental health, combining individual needs, safety, and the search for better physical fitness and psychological responses.

## Conclusions

Achieving suggested physical activity standards have been mentioned by a large body of evidence to improve immunity and enhance the deleterious effects of COVID-19 disease. Moreover, this achievement in exercise levels is especially recommended during social isolation. Guidelines addressed to the general population on each chronic disease indexed by ACSM, which recommends 150–300 min of moderate to vigorous-intensity cardiorespiratory physical activity per week and two sessions per week of muscle strength training (213) are available, as well as several protocols of HBE considering the same recommendations for the general population. Home-based platforms or video classes with physical education professionals can be useful to ensure the security and effectiveness of these protocols. In the COVID-19 pandemic situation, WHO guidelines have been recommending a similar protocol to ACSM in order to improve global health and mitigate the consequences of COVID-19. In this sense, these guidelines have considered HBE as an alternative during lockdown periods and OBE during the period that the population has access to open places with social distancing (249).

**Figure 3:**
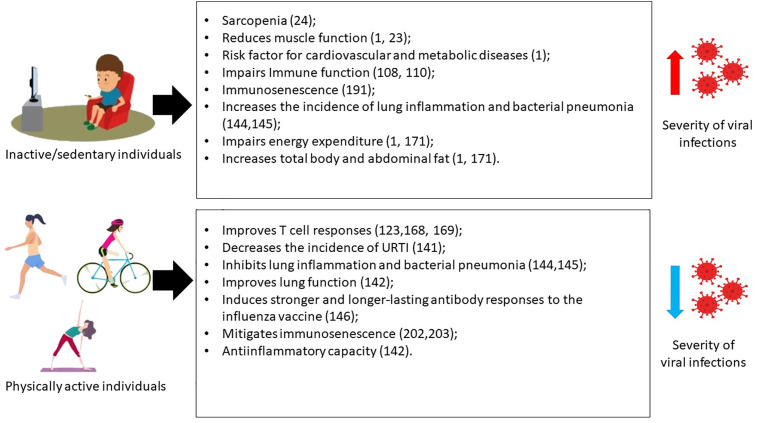
Effects of exercise and inactivity on physical health and the risk of severe disease upon viral infections. Inactivity is described as a risk factor for sarcopenia, reduced muscle function and functionality, cardiovascular and metabolic diseases, impaired immune function and immunosenescence, but also it has been associated with impairing energy expenditure, increasing total body and abdominal fat. On the other hand, physically active individuals have improved T cell responses, decreased levels of lung inflammation and bacterial pneumonia, improved lung function, stronger and long-lasting antibody responses to the influenza vaccine, decreased immunosenescence, increased anti-inflammatory capacity, and decreased upper respiratory tract infection (URTI) incidence and symptoms.

Although, exceeding WHO recommended physical activity levels during the lockdown might not be scientifically sustainable, in order to avoid high intense and prolonged exercise. Therefore, although exercise will not prevent us, directly, from SARS-CoV-2 infection, it might prevent us from developing a severe form of COVID-19, improve the immune defense, and counteract the negative effect of this disease in our immune system due to its anti-inflammatory properties shown to improve the outcome of chronic and infectious diseases (**Figure 3**). Besides, exercise is a strategy to balance the symptoms of stress due to social isolation. Moreover, physical exercise is also the most effective therapy for those who are asymptomatic or experiencing only mild symptoms, mainly vulnerable people such as the elderly and people with chronic diseases. Therefore, maintaining physical exercise levels would reduce the comorbidities of COVID-19, also minimize future complications from this disease, and should be strongly recommended to the population in this scenario.

Therefore, future studies should investigate the impact of HBE and OBE with safe social distancing on the rehabilitation of COVID-19-induced morbidities, treatment, hospitalization, and other consequences of the disease. Thus, well-designed studies are necessary to consolidate the real impact of HBE or OBE on the immune system of COVID-19 patients.

## Author Contributions

TF, AC, FS, EC, and TS conceived the study and wrote the manuscript. TF, MF, LS, WA, GA, and AC carried out the literature review, and led the draft and audit of this manuscript. TF, AC, TS, GA, LS, and FS contributed to the final revision of the manuscript. TS and FS contributed equally. In addition, the mutual and proportional commitment to support the writing, technical, and idiomatic revision of the authors is highlighted. All authors contributed to the article and approved the submitted version.

## Funding

This study was financed in part by the Coordenação de Aperfeiçoamento de Pessoal de Nível Superior - Brasil (CAPES) - Finance Code 001; the National Council for Scientific and Technological Development (CNPq) and Pernambuco Science and Technology Support Foundation (FACEPE). AC is supported by FACEPE (BFP-0102-1.11/19) and CNPq (159958/2019-9), LERS by FACEPE (IBPG-0555-4.09/18), and TOF by CAPES (88887.470105/2019-00).

## Conflict of Interest

The authors declare that the research was conducted in the absence of any commercial or financial relationships that could be construed as a potential conflict of interest.
